# Diseases of the Spinal Cord

**Published:** 1908-09

**Authors:** 


### BOOKS ON DISEASES OF THE NERVOUS SYSTEM.

**Diseases of the Spinal Cord.** By R. T. Williamson, M.D. Pp. xi., 432. London: Oxford Medical Publications. 1908.

Dr. Williamson's contributions to neurology, especially to diseases of the spinal cord, have been of the highest order, and this book embodies the results of many years' teaching and experience. The work is written to serve as a text-book, and the author is to be congratulated on having succeeded in producing an excellent one. Besides embodying his own experience, the book affords internal evidence of his wide acquaintance with the literature. In view of the great extent of this literature, he has done wisely in not attempting to give a complete bibliography in a text-book, and in adopting the more useful plan of indicating to the student the authorities most worth consulting. With regard to the book itself, a few minor criticisms may be made. The chapter on the functions of the cord is not so good as the other introductory ones, and hardly deals adequately with the matter, *e.g.* with regard to the important subject of reflex-actions, their co-ordination, etc.

The classification adopted for diseases of the cord does not work out very happily. Thus, to carry it out, Landry's paralysis and syringo-myelia come into the group of diseases causing atrophic paralysis, and disseminated sclerosis forms, with primary spastic paraplegia, a separate group, with spastic paralysis or paresis as the classifying symptom—surely a curiously assorted pair. Though one can see that in this classification the author had in his mind the dominant symptom in the majority of cases of the diseases in question, to anyone beginning the study of diseases of the cord such an arrangement is likely to be confusing, and to lead to misconception of the nature of such a disease as, for example, disseminated sclerosis. It is only fair to add that the author carefully states the difficulty with regard to Landry's disease, and points out that muscular atrophy in the typical cases is absent. We have noticed very few omissions, but in the differential diagnosis of the causation of atrophy of the small muscles of the hands we could find no mention of its occurrence in cases of cervical ribs. On pages 259 and 261 the heading at the top of the page is wrong.

We have mentioned these minor defects as they may easily be corrected in future editions, and having found fault to this extent, we hasten to add that the book is undoubtedly an excellent one, and is brought up to the most recent neurological work on the cord. The sections on pathological anatomy are good throughout, the illustrations—the large majority of which appear to be from original preparations by the author—are both well executed and well chosen, so that they materially help the text. Whilst saying that a uniformly high standard is maintained throughout the volume, it should be added that some of the sections go far beyond the ordinary text-book, and should be read by every neurologist. We should strongly advise any of our readers who require a practical and adequate guide to the diseases of the spinal cord to get this.

